# Decreased Gray Matter Volume of Cuneus and Lingual Gyrus in Schizophrenia Patients with Tardive Dyskinesia is Associated with Abnormal Involuntary Movement

**DOI:** 10.1038/s41598-018-31186-y

**Published:** 2018-08-27

**Authors:** Ting Yu, Yanli Li, Fengmei Fan, Hongbao Cao, Xingguang Luo, Shuping Tan, Fude Yang, Xiangyang Zhang, Yin Yao Shugart, L. Elliot Hong, Chiang-Shan R. Li, Yunlong Tan

**Affiliations:** 1 Peking University HuiLongGuan Clinical Medical School, Beijing, P. R. China; 2 Uniton Statistical Genomics, National Institute of Mental Health, NIH, Bethesda, 20892 USA; 3 Department of Psychiatry, Yale University School of Medicine, New Haven, CT USA; 4 Department of Psychiatry and Behavioral Sciences, Harris County Psychiatric Center, The University of Texas Health Science Center at Houston, Houston, TX USA; 5 Maryland Psychiatric Research Center, Department of Psychiatry, University of Maryland School of Medicine, Baltimore, USA

## Abstract

Tardive dyskinesia (TD) is a devastating motor disorder associated with the etiological process of schizophrenia or antipsychotic medication treatments. To examine whether cerebral morphological changes may manifest in TD, we used voxel-based morphometry to analyze high-resolution T1-weighted brain structural magnetic resonance images from 32 schizophrenics with TD (TD group), 31 schizophrenics without TD (non-TD group), and 32 healthy controls (HC group). We also assessed psychopathological symptoms with the Positive and Negative Syndrome Scale (PANSS), and TD severity with the Abnormal Involuntary Movement Scale (AIMS). We compared gray matter volumes (GMVs) among groups, and tested for correlations between GMV changes and psychopathological symptoms or TD severity. The results showed significant differences in GMV in the frontal and temporal cortices, insula and cerebellum among the three groups. Brainstem and inferior frontal and precentral gyri GMVs were significantly larger, whereas cuneus and lingual gyrus GMVs were significantly smaller in the TD group as compared to non-TD group. Further, the cuneus and lingual gyrus GMVs were positively correlated with AIMS scores in the TD group. The current results suggest that TD may be associated with the alterations in GMV that are different from that of schizophrenics without TD. Further studies are needed to confirm and to examine the functional significance of these structural findings.

## Introduction

Tardive dyskinesia (TD) is characterized by repetitive involuntary movements, usually involving the mouth, face, and tongue, and sometimes the limb and trunk musculature, as part of the etiological process of schizophrenia or side effects of antipsychotic medications. TD may occur from days to years after the initiation of antipsychotic treatment and persist even after a switch of medications to atypical antipsychotics^1,2^ or after complete withdrawal of medications^3–6^. TD reduces quality of life and disrupts a patient’s personal, social, and professional function^7^. A recent report showed that the annualized incidence of TD was 5.5% in patients treated with first-generation antipsychotics, and 3.5% in those treated with second-generation antipsychotics^8,9^. The incidence of TD with atypical antipsychotics is higher than expected, and underscores the fact that TD remains a significant clinical problem^10^. However, our understanding of the pathogenesis of TD is still lacking^11^.

Several hypotheses have been proposed to explain the development of TD, including dopamine receptor hypersensitivity, γ-aminobutyric acid (GABA) deficiency, and neuronal damage due to free radicals^12,13^. Recurring dyskinetic movements of facial and limb muscles, indistinguishable in form from tardive dyskinesia, are observed in patients with schizophrenia who have never been exposed to antipsychotics, suggesting that the intrinsic pathophysiology of schizophrenia contributes to tardive dyskinesia^4^. The leading hypothesis proposes that, as a result of chronic blocking of dopamine receptors, long-term antipsychotic treatments cause compensatory increase in dopamine receptor sensitivity^14^. On the other hand, TD symptoms may persist even after drug withdrawal, raising the issue that heightened dopamine receptor sensitivity may not account for all of the symptoms of TD.

One alternative hypothesis is that TD may be associated with structural brain changes^15^. Voxel-based morphometry (VBM) is a convenient and well-validated quantitative assessment of gray matter volumes (GMVs) by magnetic resonance imaging (MRI), frequently used to document changes in brain volumes in various neurological disorders^16^. Although not without limitations, VBM allows for an objective and automatic assessment of gray matter (GM) morphology. Few studies have focused on documenting MRI findings specific to TD, and their results were surprisingly inconsistent^17–21^. For example, Li *et al*.^19^ found that schizophrenics with TD had significantly reduced GMVs in the bilateral inferior and right superior frontal gyri in correlation with symptom severity. Sarro *et al*.^20^, however, found that TD-related volume reductions were predominantly subcortical, involving the basal ganglia and thalamus. The inconsistencies do not merely concern the anatomical locations of morphological changes; the putative correlations between morphology and TD symptom severity varied widely in the above-mentioned studies. These inconsistencies may in part be related to small sample sizes, heterogeneity in patient samples, or differences in imaging and analysis methods across the studies.

Here, we attempted to address morphological changes in TD with a more homogeneous Chinese patient sample, high-resolution imaging protocol, and standard VBM analytics. We hypothesized that TD-specific changes in GMVs would be identified in patients with schizophrenia and TD, and that the morphological changes would correlate with the clinical symptoms or severity of dyskinesia.

## Results

### Demographic and clinical characteristics

No significant differences in age, sex composition, and education were found among the three groups. Duration of illness and antipsychotic dose (CPZ) were indistinguishable between the TD and non-TD groups (all *p’s* > 0.05). However, the TD group had significantly higher PANSS Negative scores (*p* = 0.002) than the non-TD group (Table 1). No other PANSS scores were significantly different between the TD and non-TD groups.

**Table 1:** Demographic and clinical characteristics.

Variables	TD (n = 32)	Non-TD (*n* = 31)	HC (*n* = 32)	*F/T/X* ^2^	*P*
Sex (M/F)	20/12	19/12	19/13	4, 697	0.096
Age (years)	46.9 ± 10	45.7 ± 7.5	45.6 ± 7.7	0.174	0.841
Education(years)	11.5 ± 2.4	12.4 ± 2.2	11.9 ± 2.2	1.268	0.286
Duration of illness(years)	22.8 ± 10.2	22.6 ± 8.4	—	0.071	0.943
PANSS					
Positive subscore	14.4 ± 5.5	13.8 ± 5.6	—	0.405	0.687
Negative subscore	22.3 ± 5.8	17.3/6.2	—	3.311	**0.002**
General psychopathological subscore	30.9 ± 5.6	31.2 ± 7.2	—	−0.157	0.876
Total score	67.6 ± 11.7	62.3 ± 13.8	—	1.657	0.103
AIMS	8.6 ± 4.2	—	—		—
CPZ equivalents (mg)	508 ± 266	530 ± 224	—	−0.35	0.727
Haloperidol	1	0			
Amisulpride	1	0			
Clozapine	16	10			
Risperidone	4	6			
Olanzapine	8	8			
Quetiapine	2	0			
Perphenazine	0	2			
Pipotiazine	0	1			
Sulpiride	0	1			
Aripiprazole	0	2			
Lurasidone	0	1			

*p*-value indicate significant differences (*p* < 0.05) in appropriate statistical tests. PANSS: positive and negative syndrome scale; AIMS: Abnormal Involuntary Movement Scale; CPZ: chlorpromazine; TD: patients with schizophrenia and tardive dyskinesia; non-TD: patients with schizophrenia and without tardive dyskinesia; HC healthy subjects.

### Voxel-based morphometry

**Figure 1:**
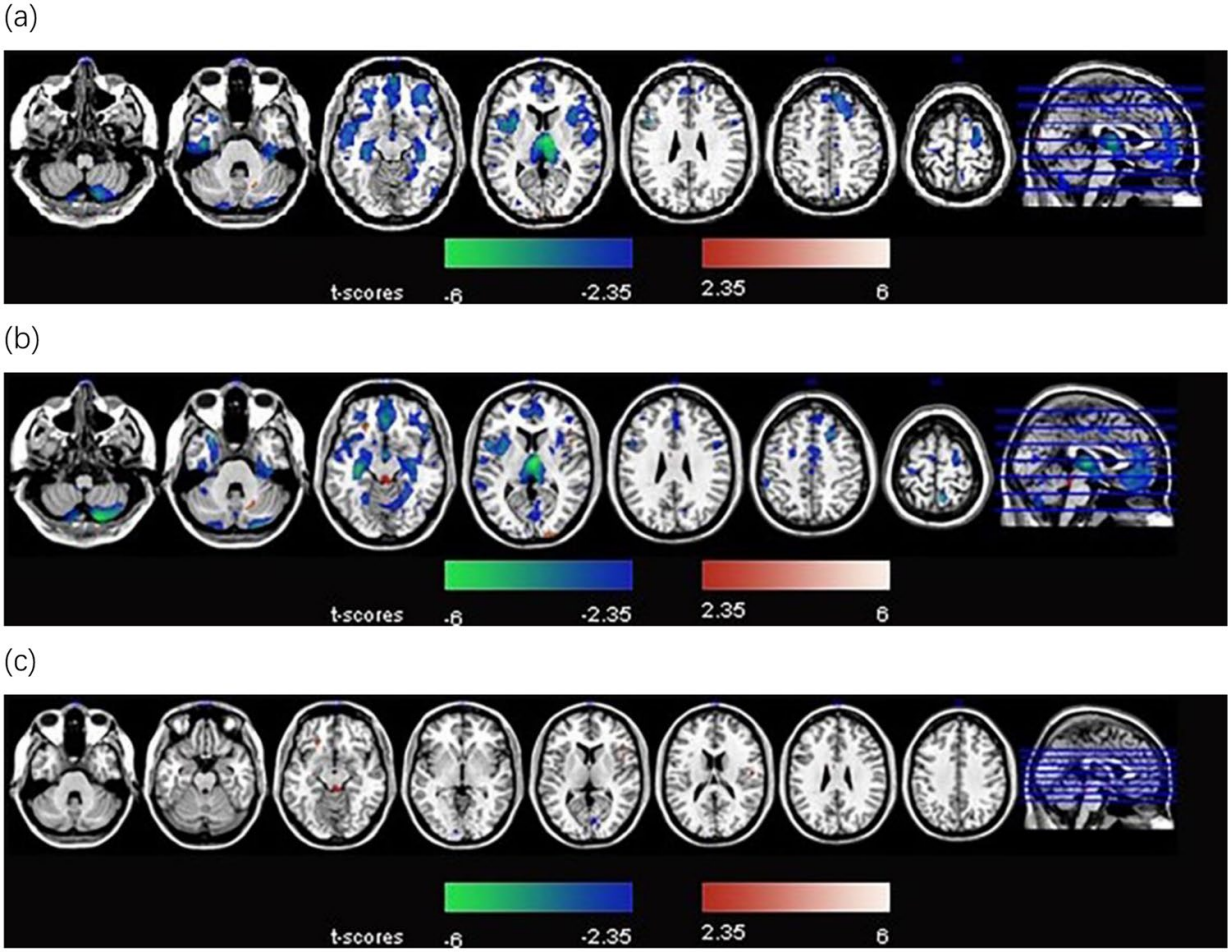
(**a**) Regions showing gray matter volume changes in schizophrenics without tardive dyskinesia (non-TD), as compared with healthy control group. Warm and cool color each represents volume increases (non-TD > control) and decreases (non-TD < control). (**b**) Regions showing gray matter volume changes in schizophrenics with tardive dyskinesia group compared with healthy control group. Warm and cool color each represents volume increases (TD > control) and decreases (TD < control). (**c**) Regions demonstrating gray matter volume changes in schizophrenics with tardive dyskinesia as compared to those without tardive dyskinesia group. Warm and cool color each represents volume increases (TD > non-TD) and decreases (TD < non-TD).

Schizophrenics both with and without TD showed significant GMV loss in multiple cortical areas including frontal and temporal cortices, insula and cerebellum, as compared with controls (*p* < 0.01). Further, the TD group demonstrated significantly greater volume reductions in the frontal areas than the non-TD group (*p* < 0.01) (Fig. 1a and b). The brainstem, inferior frontal gyrus and precentral gyrus demonstrated significant GMV increases in the TD group compared with the non-TD group; in contrast, the cuneus and lingual gyrus demonstrated significant GMV loss in the TD group relative to the non-TD group (Table 2 and Fig. 1c).

**Table 2:** Brain regions with significant volume variation in schizophrenics with tardive dyskinesia group compared to schizophrenics without tardive dyskinesia group.

Voxels size Anatomical Region	X	Y	Z	BA	Cluster size	*T*-statistic
Brain stem	0	−35	−21	—	204	3.33
Inferior Frontal Gyrus	−18	30	−14	11, 47	218	4.17
Cuneus, Lingual Gyrus	9	−77	5	17, 18, 23, 30	182	−3.22
Precentral Gyrus	54	−12	15	43, 13	101	3.1

X, Y, Z: MNI (Montreal Neurological Institute) coordinates; BA: Brodmann area; *p* < 0.05 with extent threshold of 100 voxels; *T*-statistic: peak *T* score of the cluster.

### Correlations between regional GMV changes and clinical evaluations of schizophrenia with/without TD

**Figure 2:**
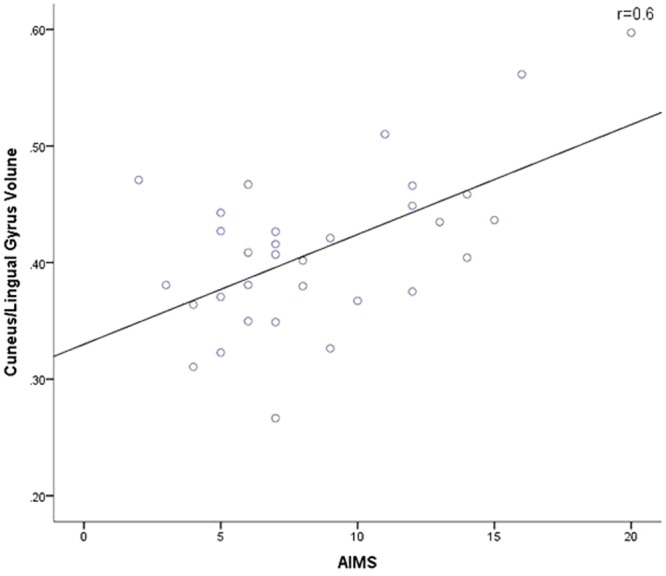
The relationship between Abnormal Involuntary Movement Scale score and gray matter changes in the cuneus or lingual gyrus in the schizophrenics with tardive dyskinesia group. The unit is cm^3^.

Among the regions that showed significant differences between the TD and non-TD groups, the cuneus and lingual gyrus changes in GMV were positively correlated with AIMS scores (*r* = 0.60, *p* = 0.001) in the TD group (Table 3 and Fig. 2), which was significant after Bonferroni correction for 4 regions of interest we tested in linear regression (*p* < 0.004). (Brodmann areas: 17, 18, 23, and 30).

**Table 3:** Relationship between clinical evaluations and gray matter volume changes derived from the comparison of schizophrenics with and without tardive dyskinesia.

Brain regions	PANSS P		PANSS N		PANSS G		PANSS T		AIMS	
	R-value	P-value	R-value	P-value	R-value	P-value	R-value	P-value	R-value	P-value
Brainstem	0.091	0.491	0.260	0.047	−0.017	0.899	0.158	0.233	0.08	0.687
Inferior Frontal Gyrus	−0.047	0.725	0.106	0.424	−0.081	0.544	−0.006	0.964	0.002	0.994
Cuneus, Lingual Gyrus	−0.015	0.912	−0.083	0.531	−0.120	0.365	−0.104	0.434	0.599	**0.001**
Precentral Gyrus	−0.071	0.595	0.033	0.803	−0.081	0.540	−0.052	0.695	0.033	0.869

PANSS: positive and negative syndrome scale; AIMS, Abnormal Involuntary Movement Scale; BA: Brodmann Area; *p*-value indicate significant differences (*p* < 0.05) in appropriate statistical tests.

## Discussion

In this study, we found that, compared with the non-TD group, the TD group had increased GMVs in the brainstem, inferior frontal gyrus, and precentral gyrus, and reduced volume in the cuneus and lingual gyrus. Furthermore, the GMV changes in the latter areas were positively correlated with AIMS scores in the TD group. We also found that patients with schizophrenia had significantly reduced GMVs in multiple cortical areas regardless of the presence of TD.

Increased GMVs in the brainstem, inferior frontal gyrus, and precentral gyrus in TD relative to non-TD may relate to the cortico-striatal-thalamic loop of information processing. Cortical motor information is relayed to the basal ganglia, which in turn sends projections back via the thalamus to the cortex. The basal ganglia output nuclei, the substantia nigra pars reticulata, and the globus pallidus all project to the prefrontal cortex via specific thalamic nuclei^22^. Thus, information from the enlarged cortical gyri is processed in the basal ganglia, which project back to the cortex via projections to the motor thalamus. A major neurotransmitter of the cortico-striatal-thalamo-cortical circuits is GABA. GABAergic interneurons are normally responsible for maintaining a balance in functions between the direct and indirect cortico-striatal-thalamic-cortical pathways^23^. We hypothesize that a decrease in GABA levels results in attenuated inhibition of thalamic neurons, which leads to cortical over activity and increased cortical input, followed by compensatory cortical hyperplasia.

Previous studies showed that decreased GABA levels are associated with TD, and an indirect GABA agonist has demonstrated therapeutic efficacy for TD^14^. Masseter motor neurons are innervated bilaterally by premotor neurons extensively distributed in the brainstem^24,25^. The corticobulbar tract mainly terminates in the premotor neuron pool of the brainstem, which receives glutamatergic afferents from the cortex^26^. It is possible that the impaired movement in schizophrenics with TD may reflect the need of more cognitive resources to perform complex activities^27^. The increased GMV may reflect a consequence of chronic TD, a compensatory process of the cortico-striatal pallidal thalamocortical circuit in response to prolonged TD symptoms.

We also found that, compared with the non-TD group, the TD group showed decreased GMV in the cuneus and lingual gyrus. Further, in TD group, cuneus and lingual gyrus GMV was in positive correlation with patients’ AIMS scores. These findings were in contrast with previous reports of decreased GMV of cuneus and lingual gyrus in schizophrenia patients as compared to healthy participants^28^ and a positive correlation of more severe GMV decreases in the right superior frontal gyrus with AIMS score. It is very difficult to explain these contrasting and paradoxical findings. Further, previous studies have reported these frontal and visual cortical abnormality as a characteristic of schizophrenia^29^. Thus, the current findings remain to be confirmed and whether these structural brain changes are specific to TD remains to be investigated.

It is also worth noting that the cases included in Gupta’s mega-analysis^29^ were mostly medicated. Typical antipsychotics have been reported to be associated with enlarged GMV in schizophrenic patients^30^. However, the atypical antipsychotic clozapine was associated with a decrease in caudate size in schizophrenia patients previously treated with typical antipsychotics^17^. Here, the medication status of the TD and non-TD groups are indistinguishable. Therefore, the current GMV findings may not be due to medication treatment. In the current study TD and non-TD patients were indistinguishable in their medication treatments.

The leading models of the etiology of TD include dopamine receptor supersensitivity, GABA depletion, cholinergic deficiency, neurotoxicity, oxidative stress, changes in synaptic plasticity, and defective neuro-adaptive signaling. Other studies have suggested that TD may be related to reduction in GM volumes^19,20^, which would be consistent with our general observations herein. Previous studies described decreased GMV in the basal ganglia in TD^20^, which we failed to observe in the current study. There are a few potential explanations for this discrepancy. Firstly, the sample sizes of these two studies are moderate. Inconsistencies between small and moderate sized studies are not surprising in light of the wide diversity in the findings regarding GM abnormalities reported by small and moderate sized studies. Secondly, this diversity is probably largely due to the heterogeneity of the condition across subjects. The subjects in the study by Sarro *et al*. are Spanish, while those subjects in our study are Chinese. Thirdly, the methods of analyzing images are different. The data of the study by Sarro *et al*. were analyzed with FSL-VBM, while our study used VBM based on MATLAB. More studies with larger sample sizes are definitely warranted to resolve these discrepancies. Furthermore, severity of TD is associated with the severity of negative symptoms in schizophrenia. It is plausible that both dopamine blocking medication and the pathophysiology of schizophrenia itself contribute to TD^4^.

Several limitations should be considered when interpreting the results of the present study. The data are cross-sectional; therefore, the dynamic changes in brain structure during the progression of TD could not be assessed. The small sample size is another limitation of this study. A meta-analysis integrating multiple studies to generate a more comprehensive understanding of TD and grey matter variations is warranted. In particular, longitudinal studies would provide deeper insight into the relationship of cerebral GMV and the pathophysiology of TD. Finally, changes in functional activity and connectivity should be assessed along with cerebral morphometry to better understand the neural bases of TD.

In sum, although the availability of second generation antipsychotic medications has reduced the incidence and severity of TD, it remains a potentially severe side effect of the treatment for schizophrenia. The current results suggest that TD may be associated with the alterations in GMV that are different from that of schizophrenics without TD. Further studies are needed to validate this finding. These findings may facilitate new studies on the neural markers of this debilitating condition.

## Methods

### Participants

Thirty-two (20 men and 12 women, age: 46.9 ± 10.0 years) right-handed schizophrenics with TD (TD group), thirty-one schizophrenics (19 men and 12 women, age: 45.7 ± 7.3 years) without TD (non-TD group), and thirty-two (19 men and 13 women, age: 48.8 ± 7.9 years) healthy controls (HC group) were recruited for this study (Table 1). All patients were recruited from the inpatient services at Beijing HuiLongGuan Hospital. All schizophrenia patients met the following inclusion criteria: 1) age 18–60 years; 2) meeting the Diagnostic and Statistical Manual of Mental Disorders (Fourth Edition, Text Revision) (DSM-IV-TR) criteria for schizophrenia after a structured clinical interview for DSM-IV-TR Axis I Disorders, Patient’s Version; 3) diagnosis with schizophrenia for at least 5 years; 4) having received stable doses of antipsychotics for at least 6 months before the study. The HC group comprised healthy residents drawn from the same period. Subjects were excluded if they met one of the following criteria: 1) a DSM-IV Axis I diagnosis other than schizophrenia; 2) serious, acute, unstable and/or significant and untreated medical illness (e.g. infection, diabetes, and uncontrolled hypertension); 3) a history of brain trauma or neurological disease; 4) pregnant or breast-feeding; 5) taken neurotrophic or free radical metabolism drugs within the 12 weeks prior to participation.

TD was diagnosed using Schooler and Kane’s Research Diagnostic Criteria (1982)^31^, and its severity was assessed using the Abnormal Involuntary Movements Scale (AIMS)^32^. The TD clinical ratings were performed by two experienced psychiatrists who were clinically trained to consistently perform AIMS testing as part of the general hospital training procedures, although they were not specifically trained for this study. In addition, patients with TD were re-evaluated for AIMS score by the same psychiatrist at least one month later, and were diagnosed with TD only if both evaluations yielded positive results. Psychopathology was assessed using the Positive and Negative Syndrome Scale (PANSS)^33^. Both scales had intra class correlation coefficients ≥0.75. This study was approved by the Ethics Review Board (IRB) of the Beijing HuiLongGuan hospital. All participants gave written informed consent before participating in the study. All patients were recruited from the inpatient services at Beijing HuiLongGuan Hospital according to a cross-sectional naturalistic design. All study procedures followed this research protocol. We confirmed that all research was performed in accordance with the relevant guidelines.

### Data acquisition

Structural MRI data were acquired on a 3.0-Tesla MR scanner (Trio system; Siemens, Erlangen, Germany) using a T1-weighted (T1W) sagittal 3D-MPRAGE (magnetization prepared rapid acquisition with gradient echo) sequence: echo time (TE) = 2.6 ms; inversion time (TI) = 800 ms; repetition time (TR) = 1,600 ms; flip angle (FA) = 9°; field of view (FOV) = 256 mm × 224 mm; matrix size = 512 × 448; slice thickness = 1 mm; voxel dimension = 1 mm × 1 mm × 1 mm. T2-weighted images (T2WI) were acquired using a turbo-spin-echo (TSE) sequence: 20 axial slices, thickness/gap = 5/1.5 mm, matrix = 512 × 416, TR = 4,140 ms, TE = 92 ms, FA = 150°, FOV = 187 mm × 230 mm.

### VBM analysis

Individual high-resolution T1-weighted data were analyzed using the VBM8-Toolbox (http://dbm.neuro.uni-jena.de/vbm) technique^34^, implemented with Statistical Parametric Mapping (SPM8) (http://www.fil.ion.ucl.ac.uk/spm/software/spm8), and executed in MATLAB 2010 (https://www.mathworks.com/products/matlab.html). First, data from each participant were carefully checked three times for any scanner artifacts, motion problems or gross anatomical abnormalities. Then a noise reduction procedure was performed on each participant’s native space T1W structural image using a spatial-adaptive non-local-means denoising filter.

For image preprocessing, T1 images were first segmented into GM, white matter (WM) and cerebrospinal fluid (CSF) and normalized to an Montreal Neurological Institute (MNI) template using the unified standard segmentation option in SPM8^35^. The total GMV of each voxel was obtained through modulation by multiplying the GM concentration map by the non-linear determinants derived from the spatial normalization step. All normalized, segmented, and modulated MNI standard space MR images were then visually checked for quality and smoothed using a Gaussian filter with a full-width at half-maximum smoothing kernel of 8 mm. Total intracranial volume (TIV) was calculated as the sum of GM, WM and CSF volumes. Finally, after image smoothing, the GM images were entered into statistical modeling to obtain cluster sizes for each group.

### Statistical analysis

ANOVAs were used to compare the age and education among the three groups. Non-parametric tests were used to compare the sex composition among the three groups. T-tests were used to compare the PANSS scores (Total, Negative, Positive, and General), medication in chlorpromazine (CPZ) equivalents, and duration of illness between the patient groups. Voxel-wise GMV differences among the three groups were investigated using ANCOVA, with age, sex, education and TIV as covariates. To avoid possible edge effects around the margin between different tissue types, all voxels with a GM probability value lower than 0.2 were excluded. Statistical inferences were made with a voxel-level statistical threshold (*p* < 0.01), after applying an extent threshold (*p* < 0.001 uncorrected) with a minimum cluster size of 100 voxels (with voxel size equal to 1 mm^3^)^36,37^. Then we performed correlation analyses to explore the relationship between regions with nominal TD vs. non-TD differences, and that between GMV differences and severity of TD symptoms as assessed by AIMS in the TD group, again using age, sex, education and CPZ as covariates. Volume size, clinical symptoms (PANSS Total), and other clinical parameters were also explored. GMV group comparisons were performed in SPM8 and the correlation analyses between volume size of brain regions and AIMS were performed using SPSS 20.0 (IBM, USA).
